# AI-Generated Exposure Hierarchies for Body Dysmorphic Disorder: Proof-of-Concept Study

**DOI:** 10.2196/98393

**Published:** 2026-11-03

**Authors:** Geneva K Jonathan, Adam C Jaroszewski, Heyli T Arcese, Natasha H Bailen, Ivar Snorrason, Anne Chosak, Jennifer Ragan, Susan Sprich, Jessica Rasmussen, Emily E Bernstein, Sabine Wilhelm

**Affiliations:** 1 Department of Psychiatry, Massachusetts General Hospital, 185 Cambridge Street, Suite 2000, Boston, MA, 02114, United States, 1 6177246146; 2 Department of Psychiatry, Harvard Medical School, Boston, MA, United States; 3 iris labs, Cambridge, MA, United States

**Keywords:** body dysmorphic disorder, exposure therapy, cognitive behavioral therapy, AI, large language models, treatment planning, clinical decision support, insight, mental health, digital health

## Abstract

**Background:**

Body dysmorphic disorder (BDD) is a chronic, impairing condition for which cognitive behavioral therapy, including exposure and response prevention (ERP), is the first-line treatment, but few clinicians are trained in BDD-specific ERP. Constructing personalized exposure hierarchies is time-intensive and requires clinical expertise, particularly for patients with absent insight who may hold appearance beliefs with delusional conviction. Generative AI (GenAI)–powered chatbots, such as ChatGPT, have shown utility for clinical content, but their capacity to produce safe, appropriate ERP hierarchies for BDD has not been examined.

**Objective:**

This study evaluated the feasibility of using ChatGPT (GPT-5) to generate ERP hierarchies for BDD, assessed the influence of clinical and demographic characteristics on output quality, benchmarked AI-generated hierarchies against those created by doctoral-level BDD specialists, and tested whether blinded experts could distinguish AI-generated from expert-generated hierarchies.

**Methods:**

GPT-5 generated 10-item graded exposure hierarchies for 72 simulated vignettes that varied by body area, insight level (good, limited, or absent), symptom specificity, age, and gender. Expert clinicians independently generated hierarchies for a subset of 18 vignettes. Two researchers rated all GPT-5 hierarchies for task completion and information integration. Three BDD expert clinicians, blinded to the hierarchy source (expert vs GPT-5), rated all 90 hierarchies for relevance, specificity, variability, safety, and overall quality. After unblinding, raters attempted to identify the AI-generated hierarchy in the 18 matched pairs.

**Results:**

GPT-5 generated complete hierarchies for 69 of 72 (95.8%) vignettes. Blinded experts rated GPT-5 hierarchies highly across all dimensions (mean scores ranging from 4.39, SD 0.26 to 4.90, SD 0.16 out of 5), with no significant differences from expert-generated hierarchies except for variability, where experts scored modestly higher (*P*=.02; *r*=0.24). After unblinding, raters correctly identified AI-generated hierarchies only 31.5% (17/54) of the time, significantly below chance (*P*=.009); this below-chance result was mostly due to one rater, and no rater performed above chance. Insight level was the most consistent predictor of quality: absent-insight hierarchies received significantly lower safety (*P*<.001; *r*=0.73) and overall quality ratings (*P*=.01; *r*=0.43). This pattern was not unique to GPT-5; safety ratings for expert-generated hierarchies also declined significantly by insight level (*P*=.03). Miscalibration of exposure difficulty, particularly for low- and absent-insight cases, was the most common rater concern (30/43, 69.8% comments), disproportionately directed at GPT-5 hierarchies.

**Conclusions:**

GPT-5 generated ERP hierarchies for BDD that blinded experts rated as comparable to clinician-created hierarchies and could not reliably distinguish them from clinician-created hierarchies. However, hierarchies for patients with absent insight received lower safety and quality ratings from both AI and expert sources, and qualitative comments suggested AI-generated hierarchies were less well calibrated to delusional-level presentations. GenAI thus shows promise as a clinician-facing aid for ERP hierarchy generation, but these findings underscore the need for clinician oversight, especially for patients with delusional conviction.

## Introduction

Body dysmorphic disorder (BDD) is characterized by distressing preoccupation with perceived defects or flaws in physical appearance that are not observable or appear only slight to others, and is accompanied by repetitive behaviors such as mirror checking, reassurance seeking, and camouflaging [1]. The disorder affects an estimated 1.7% to 2.4% of the general population [2,3], follows a chronic course when untreated [4], and is associated with significant functional impairment, social withdrawal, and elevated risk for suicidal ideation and behavior [5,6].

Cognitive behavioral therapy (CBT) with exposure and response prevention (ERP) is the gold-standard, evidence-based treatment for BDD [7,8]. Treatment is organized around an exposure hierarchy in which patients progressively approach appearance-related triggers (eg, social situations, specific activities, and previously avoided contexts) to test and challenge appearance-related beliefs while refraining from safety behaviors such as mirror checking, camouflaging, and comparing themselves to others [9]. Constructing an effective hierarchy requires the clinician to translate each patient’s preoccupations, cognitive distortions, and avoidance behaviors into a set of exercises that are clinically appropriate, graded in difficulty, and safe to implement.

Access to this treatment, however, remains severely limited. Few clinicians are trained in BDD-specific ERP, and most individuals with BDD present first to dermatological or cosmetic settings rather than to mental health providers [10,11]. Efforts to expand the treatment workforce through virtual training programs and to deliver interventions through digital platforms have shown early potential [12-14], but these approaches are commonly challenged by the need to generate personalized exposure content tailored to each patient. Hierarchy development is time-consuming even for experienced clinicians and presents a particular challenge for clinicians less familiar with BDD and for patients attempting self-guided treatment [15,16].

Beyond these access barriers, hierarchy development for BDD poses a distinct clinical challenge related to the role of patient insight. Insight, the degree to which individuals with BDD recognize that their appearance beliefs are inaccurate, spans a broad continuum from good to absent (ie, delusional conviction) [17,18]. The *DSM-5* (*Diagnostic and Statistical Manual of Mental Disorders, Fifth Edition*) captures this variation with an insight specifier distinguishing good or fair, poor, and absent insight/delusional beliefs [1]. BDD is classified as an obsessive-compulsive and related disorder because it shares core phenomenology and a first-line treatment (ERP) with obsessive-compulsive disorder (OCD), yet differs strikingly in insight distribution. Whereas most patients with OCD demonstrate excellent or good insight and only 2% hold delusional beliefs, approximately 70% of individuals with BDD show limited or absent insight, and roughly one-third are fully delusional [19]. Absent insight in BDD has been associated with greater symptom severity, increased suicidal ideation [20], and, in some studies, poorer response to CBT [18,21]. Patients with absent insight may view exposure exercises as irrelevant to what they perceive as a genuine physical defect, potentially requiring more thorough discussion of exposure rationale and careful calibration of exposure intensity [17,22]. Because patients with limited or absent insight may be less prepared for the distress that exposures can provoke, a concern that is heightened by the elevated rates of suicidal ideation in BDD [20], any tool designed to assist with ERP hierarchy generation must be evaluated for its capacity to produce appropriate and safe recommendations across this full range of insight.

One class of tools with potential to address these challenges is generative AI (GenAI). GenAI describes a specific class of AI models that can generate *new* content (eg, text and images) that resembles what humans produce. Large language models, which power popular chatbots, such as ChatGPT (GPT-5), are a subtype of GenAI [23]. A growing body of research has explored the potential of GenAI to address gaps in mental health care [24,25]. GenAI systems such as GPT-5 (OpenAI, Inc), Claude (Anthropic), and Gemini (Google), which generate contextually relevant text in response to natural language prompts, have been applied across clinical tasks including education and training, symptom assessment, and treatment delivery [24,25]. Among clinician-facing applications, initial proof-of-concept studies suggest that general-purpose GenAI can generate reasonably acceptable evidence-based treatment recommendations with minimal prompt engineering, though outputs are typically rated below expert quality [26-28]. GenAI clinical training materials have been rated comparably to expert-created versions on measures of validity and appropriateness, though they tend to lack nuance and atypical presentations that characterize real-world clinical work [29]. Although GenAI outputs have been considered feasible and safe, accuracy and clinical appropriateness require ongoing improvement, and no high-quality evidence yet supports the use of general-purpose models for delivering psychological treatments [24,25]. These findings point to the importance of rigorous, blinded expert evaluation of AI-generated clinical content, particularly for disorders where clinical complexity may pose additional challenges.

One potential clinical application of GenAI is the generation of behavioral treatment plans. Recently, our team conducted the first proof-of-concept study examining whether GPT-4 could generate ERP hierarchies for OCD [28]. Blinded expert clinicians rated the AI-generated hierarchies as generally acceptable and comparably safe to expert-generated hierarchies, though expert versions were rated significantly higher on appropriateness, specificity, and usefulness. Raters also correctly identified AI-generated hierarchies at rates well above chance (67%‐94%), suggesting that the outputs, while clinically adequate, were distinguishable from expert work. This study extends this approach to BDD, which poses a more stringent test of AI-generated hierarchy quality. Because limited or absent insight is far more common in BDD than in OCD, the feasibility demonstrated for OCD, where most patients retain sufficient insight to engage with the exposure rationale, does not establish that the same approach will succeed for BDD, where delusional conviction may require fundamentally different calibration of exposure difficulty. This makes BDD an informative test case: success would suggest broader applicability of GenAI-assisted hierarchy generation, while difficulty, particularly for limited- or absent-insight cases, would help define the clinical boundaries within which such tools can be safely deployed. We examined whether GPT-5 could generate clinically appropriate, safe, and specific ERP hierarchies for BDD across systematically varied case presentations. We assessed the influence of body area of concern, insight level, symptom specificity, patient age, and patient gender on the quality of AI-generated hierarchies, with particular interest in whether hierarchies for cases with absent insight would receive lower safety or quality ratings than those for cases with better insight. Performance was benchmarked against hierarchies created by doctoral-level BDD specialists, and we tested whether blinded BDD expert clinicians could reliably distinguish AI-generated from expert-generated hierarchies.

## Methods

### Ethical Considerations

This study was reviewed by the Mass General Brigham Institutional Review Board (IRB) and determined not to constitute human subjects research under 45 CFR 46 regulations [30]; consequently, IRB approval was not required. No patient data were collected; all hierarchies were generated from simulated vignettes.

### Hierarchy Generation

We used GPT-5 to generate 10-item graded exposure hierarchies in response to systematically varied simulated patient vignettes. GPT-5 was the most recent publicly available OpenAI model at the time of data collection. Data were collected between August and September 2025. All vignettes were submitted through Mass General Brigham’s institutional Microsoft Azure OpenAI Service (Azure AI Foundry) deployment, using GPT-5 (version gpt-5-chat, released August 7, 2025). To ensure outputs were independent, each vignette was sent as a separate request containing only the standardized prompt, with no conversation history shared across requests; because each request is stateless, the consumer ChatGPT app’s memory, custom instructions, and personalization features did not apply. Generation parameters were set to a temperature of 0.7, top *P* of .95, and a maximum of 16,384 output tokens.

Also, we used a “zero-shot” approach, where no task-specific fine-tuning, no example hierarchies, and no additional prompting after the initial prompt were used to influence how or what GPT-5 produced. All vignettes were submitted from the same account. Clinicians specializing in BDD diagnosis and treatment at the Mass General Brigham Center for OCD and Related Disorders drafted all vignettes, which were submitted via the GPT-5 API. Four GPT-5 outputs were incorrectly entered into the study database during transcription, resulting in 4 vignettes being omitted from the initial review set. These hierarchies were regenerated from their original vignettes and added to the review.

Following established best practices for prompt engineering [31], we constructed vignettes with 4 components: (1) situating context, (2) role specification for the model, (3) explicit task instructions, and (4) desired output structure. The prompt was intentionally minimal, without specifying quality criteria such as variability or required exposure modalities to evaluate the model’s out-of-the-box performance. The standardized template read:

*I am a mental health provider creating a treatment plan. Please respond as if you are an expert in Exposure and Response Prevention (ERP) treatment for Body Dysmorphic Disorder (BDD). Please create a 10-item graded exposure hierarchy for a patient who is preoccupied with [body area]. They believe [specific belief about the body area], which they believe makes their [body area] look disgusting, and fear others will reject them because of this flaw. The corresponding safety behavior(s) is(are) [specific behaviors]. They have [level of insight]. The patient identifies as a [gender] and is [age] years old.*

Recognizing that vignette characteristics influence GenAI performance [31], we maintained consistent formatting while systematically manipulating 5 variables: body area of primary concern (hair, skin, and nose), insight level (good, limited, and absent; the limited level corresponds to the *DSM-5* “poor insight” specifier), symptom description specificity (low and high), patient age (adolescents of 15 years and adults of 40 years), and patient gender (woman and man). Low-specificity vignettes contained a brief description of the patient’s preoccupation, core belief, and safety behaviors (eg, “They believe their acne scarring is permanent and highly visible...The corresponding safety behaviors are mirror checking, camouflaging, comparison, and avoidance”). High-specificity vignettes expanded on the same clinical features with detailed descriptions of the frequency, duration, and contextual triggers of each safety behavior. The complete factorial design (3 body areas×3 insight levels×2 specificity levels×2 ages×2 genders) yielded 72 unique vignettes. Complete examples of a low-specificity vignette and a high-specificity vignette are provided in Multimedia Appendix 1. We limited gender to 2 categories (woman and man) to keep the total number of vignettes manageable; nonbinary and transgender identities were not represented in the vignettes.

### Expert (Human) Hierarchies

To establish a benchmark for comparison, 2 expert clinicians (NHB and IS) each independently constructed 9 hierarchies (18 total), one per cell of the 3 (body area) × 3 (insight level) × 2 (specificity) design, with age and gender counterbalanced across cases. A third expert clinician (JR) then reviewed all 18 hierarchies; the refined versions were submitted directly for blinded rating. Experts were given the same vignette text submitted to GPT-5 and were asked to produce a 10-item graded exposure hierarchy. They were not informed that their hierarchies would be compared against AI-generated output, and no additional clinical information beyond the vignette was provided.

### Data Analysis

This cross-sectional proof-of-concept study was reported in accordance with the STROBE (Strengthening the Reporting of Observational Studies in Epidemiology) guideline for cross-sectional studies [32] (Checklist 1). All analyses were conducted in R (version 4.4.2; R Foundation for Statistical Computing). Because clinician ratings were highly negatively skewed and showed ceiling effects, with 258 (95.6%) to 269 (99.6%) of 270 ratings falling between 4 and 5, residuals from parametric models were not normally distributed. We therefore used nonparametric tests throughout: Kruskal-Wallis tests (in place of ANOVA) for 3-level predictors, Wilcoxon rank-sum tests (in place of *t* tests) for 2-level predictors and pairwise comparisons, and rank-biserial correlations (*r*) as effect size estimates. Pairwise comparisons following significant Kruskal-Wallis tests were corrected using the Holm method. Fisher exact tests were used for categorical outcomes. Binomial tests evaluated whether unblinding accuracy differed from chance (50%). Conventional intraclass correlation coefficients were planned but proved uninterpretable due to ceiling effects (“Results” section). For both researcher and clinician ratings, interrater agreement was therefore assessed using Gwet agreement coefficient (AC2) with ordinal weights [33], a chance-corrected statistic robust to skewed marginal distributions, supplemented by descriptive consensus metrics (percent exact agreement, within-one-point agreement, and the proportion of hierarchies receiving convergent ratings).

### Initial Review

Two doctoral-level psychologists (GKJ and ACJ) independently evaluated all 72 AI-generated hierarchies on two dimensions: (1) task completion and (2) input information integration. Task completion was coded categorically: “not at all” indicated that GPT-5 failed to produce any usable suggestions, “complete” indicated that the model generated 10 scenarios meeting the operational definition of exposure with response prevention, and “partial” described all remaining responses. Input information integration was rated on a 5-point scale (1=“not at all” to 5=“completely”) assessing the degree to which generated hierarchies reflected the clinical information provided in the vignette (eg, correct body area, age, gender, insight level, and safety behaviors). This metric evaluated content matching rather than clinical quality. Fisher exact tests examined whether clinical vignette characteristics (eg, age and area of concern) predicted task completion, and Kruskal-Wallis or Wilcoxon rank-sum tests examined whether vignette characteristics predicted information integration ratings.

### Blinded Review

Three doctoral-level psychologists (SS, AC, and J Rasmussen) with expertise in BDD assessment and ERP delivery served as independent, blinded evaluators. These raters were blind to both the study hypotheses and the source of each hierarchy (AI vs expert). They received the following cover story: our team was piloting training programs to expand ERP delivery capacity for BDD and sought external expert feedback on trainee-generated work products. Raters received written instructions and the rating rubric but did not undergo a formal calibration procedure (eg, anchor examples, training round, or scoring discussions) prior to independent rating.

Raters evaluated all 90 hierarchies (72 AI-generated and 18 expert-generated) on five dimensions, each assessed on a 5-point scale, from 1=“strongly disagree” to 5=“strongly agree”: (1) relevance (clinical fit between suggested ERP exercises and patient symptoms), (2) specificity (actionability and clarity of suggestions), (3) variability (coverage of the expected range of exposure difficulty; raters were informed that all hierarchies were intended to contain 10 items and that they should not penalize based on the number of ERP suggestions), (4) safety (degree to which suggestions meet ethical and safety standards), and (5) overall quality. Ratings were collected via a REDCap (Vanderbilt University) survey. These rating dimensions were rationally derived by the study team for use in Bernstein et al [28] and were retained here to enable cross-study comparison. The complete item wording as presented to raters is provided in Multimedia Appendix 1.

The raters split their time across the hospital clinic (mean 28.33%, SD 17.56%), private practice (mean 30%, SD 34.64%), and other settings including administration and training (mean 38.33%, SD 33.29%). They were on average 22.33 (SD 8.50) years post–terminal degree and reported treating 11 to 50 patients with BDD with ERP over the course of their careers. They used ERP in the treatment of 85% to 100% of their patients with BDD (mean 95%, SD 8.66%) and currently carried caseloads in which 0% to 20% of patients had a BDD diagnosis.

Wilcoxon rank-sum tests compared GPT-5–generated and expert-generated hierarchies on each dimension. Kruskal-Wallis and Wilcoxon rank-sum tests then examined whether vignette characteristics (body area, insight level, symptom specificity, age, and gender) predicted ratings of the AI-generated hierarchies specifically.

Expert raters’ qualitative comments were independently coded by 2 authors (GKJ and ACJ) into 4 categories: too difficult for the patient’s level of insight (n=14), insufficiently challenging (n=16), missing a key exposure activity (n=7), or other (n=6); discrepancies were resolved by consensus. An exploratory Fisher exact test examined whether hierarchy source (GPT-5 vs expert) predicted the likelihood of receiving at least one comment indicating that the hierarchy was miscalibrated in terms of difficulty (ie, too difficult or insufficiently challenging) given aspects of the clinical case presentation (eg, insight/delusionality), at the hierarchy level (n=90).

### Unblinding

Following the completion of the blinded ratings, evaluators were informed that some hierarchies had been generated by AI while others were created by experts. They then reviewed 18 matched pairs, each consisting of 1 AI-generated and 1 expert-generated hierarchy derived from an identical clinical vignette. For each pair, raters attempted to identify which hierarchy was AI-generated and provided a confidence rating (0%‐100%). The AI-generated hierarchy always appeared at the top of the screen; this was not counterbalanced. Binomial tests evaluated whether overall and per-rater accuracy differed from chance (50%), and a Wilcoxon rank-sum test examined whether confidence ratings differed between correct and incorrect identifications.

## Results

### Interrater Reliability

Ratings showed a restricted range at the upper end of the scale, with 95.6% (258/270) to 99.6% (269/270) rated either 4 or 5, although the proportion at the true ceiling (5) varied from 46.7% (126/270) for variability to 90.0% (243/270) for safety. AC2 values indicated almost perfect agreement across all clinician-rated dimensions: safety (0.975, 95% CI 0.965‐0.985), relevance (0.951, 95% CI 0.934‐0.968), specificity (0.923, 95% CI 0.904‐0.943), overall quality (0.901, 95% CI 0.880‐0.923), and variability (0.858, 95% CI 0.834‐0.881; all *P*<.001). For researcher ratings, agreement was similarly strong for task completion (AC2=0.985, 95% CI 0.953‐1.000) and information integration (AC2=0.929, 95% CI 0.892‐0.967). Pairwise within-one-point agreement ranged from 94.4% (255/270) to 99.3% (268/270); however, given that the vast majority of ratings were already 4 or 5, this level of agreement would be expected largely by default. Among hierarchies where at least one rater assigned a score of 4 or below, convergence was highest for variability (53/85, 62.4% of flagged hierarchies had ≥2 raters assigning ≤4) and lowest for safety (1/26, 3.8%), consistent with greater rater discrimination on the dimension with the most distributional spread.

### Initial Review

GPT-5 generated complete responses for 69 of 72 (95.8%) vignettes, with the remaining 3 hierarchies rated as partially complete. No vignette resulted in a complete failure to generate relevant exposure suggestions. GPT-5 also integrated the clinical information provided in the vignettes to a high degree (mean 4.66, SD 0.42, on a 1‐5 scale). No hierarchy received a mean rating at or below the scale midpoint on any dimension.

Vignette characteristics were largely unrelated to task completion (all Fisher exact test *P*>.75; Table 1). For information integration, level of insight was the only significant predictor, *χ*^²^_2_=12.8; *P*=.002, with post hoc comparisons indicating that hierarchies generated for patients with absent insight received lower information integration ratings (mean 4.42, SD 0.48; *P*=.002; *r*=0.49) than those generated for patients with limited insight (mean 4.73, SD 0.33; *P*=.04; *r*=0.34) or good insight (mean 4.83, SD 0.32; *P*=.002; *r*=0.49). Area of concern, symptom specificity, age, and gender were not significant predictors of information integration (all *P*>.24).

**Table 1.** Predictors of task completion and information integration^a^.

Predictors	Task completion (*P* value)^b^	Information integration	
		Test statistics	Rank-biserial correlation (*r*)^c^
Area of concern	.76	2.5 (2)^d^; *P*=.29	0.14
Symptom specificity	>.99	742^e^; *P*=.25	0.14
Level of insight	.77	12.8 (2)^d^; *P*=.002	0.49
Age	>.99	627.5^e^; *P*=.80	0.03
Gender	>.99	606^e^; *P*=.61	0.06

a AI-generated hierarchies (n=72) were rated by 2 researchers.

b Task completion was assessed via Fisher exact test due to near-ceiling performance (95.8% were rated as complete).

c *r* is the rank-biserial correlation (effect size for the largest pairwise comparison for 3-level predictors).

d Information integration was assessed via Kruskal-Wallis tests; the test statistic is χ²-distributed and is reported as χ²(df).

e Information integration was assessed via Wilcoxon rank-sum tests (2-level predictors).

### Blinded Review

Blinded clinician ratings are summarized in Table 2. Both expert- and AI-generated hierarchies received high ratings across all 5 dimensions. We did not detect significant differences in relevance, specificity, safety, or overall quality (all *P*>.38). However, expert-generated hierarchies were rated as modestly more variable than GPT-5 hierarchies (*r*=0.24).

**Table 2.** Blinded ratings of hierarchies^a^.

Dimensions	AI-generated (n=72), mean (SD; range)	Expert-generated (n=18), mean (SD; range)	Wilcoxon rank-sum test^b^	Rank-biserial correlation, *r*
Relevance	4.81 (0.23; 3.67‐5.00)	4.80 (0.26; 4.33‐5.00)	660; *P=.*89	0.01
Specificity	4.70 (0.28; 3.67‐5.00)	4.69 (0.24; 4.33‐5.00)	692; *P=*.64	0.05
Variability	4.39 (0.26; 4.00‐5.00)	4.54 (0.26; 4.00‐5.00)	438; *P=*.02	0.24
Safety	4.90 (0.16; 4.33‐5.00)	4.89 (0.20; 4.33‐5.00)	647; *P=*.99	0.001
Overall	4.66 (0.26; 3.67‐5.00)	4.61 (0.26; 4.33‐5.00)	727; *P=*.39	0.09

a Ratings were averaged across 3 blinded clinician raters for each hierarchy on a 1-5 scale.

b Wilcoxon rank-sum tests were used due to nonnormal distributions.

We next examined whether vignette characteristics predicted the quality of AI-generated hierarchies (Table 3). Level of insight was the most consistent predictor, significantly influencing both safety (*P*<.001) and overall quality ratings (*P*=.01). Hierarchies generated for simulated patients with absent insight received the lowest safety ratings, with large effects relative to both limited insight (*r*=0.54; absent vs limited) and good insight (*r*=0.73; absent vs good). Overall quality showed a similar gradient, driven primarily by lower ratings for absent-insight vignettes relative to good-insight vignettes (*r*=0.43). Two other effects reached significance: symptom specificity predicted specificity ratings, and area of concern predicted variability ratings, but these were small in magnitude (all *r*<0.24). Age and gender were unrelated to ratings on any dimension.

**Table 3.** Predictors of ratings for AI-generated hierarchies^a^.

Predictors	Relevance	Specificity	Variability	Safety	Overall
Area of concern, χ² (df) ^b^	2.5 (2); *P*=.29	2.4 (2); *P*=.31	6.3 (2); *P*=.04	1.3 (2); *P*=.52	0.7 (2); *P*=.71
Symptom specificity, *W*^c^	588; *P*=.45	479.5; *P*=.04	763; *P*=.16	589.5; *P*=.41	679; *P*=.71
Level of insight, χ² (df)^b^	0.1 (2); *P*=.95	4.7 (2); *P*=.09	2.8 (2); *P*=.25	31.5 (2); *P*<.001	9.3 (2); *P*=.01
Age, *W*^c^	674.5; *P*=.74	611.5; *P*=.66	776.5; *P*=.12	635.5; *P*=.86	625; *P*=.78
Gender, *W*^c^	674.5; *P*=.74	640.5; *P*=.93	779.5; *P*=.11	564.5; *P*=.24	707.5; *P*=.47

a Ratings were averaged across 3 blinded clinician raters per hierarchy (n=72).

b Kruskal-Wallis tests were used for 3-level predictors; the test statistic is χ²-distributed and is reported as χ²(df).

c Wilcoxon rank-sum tests (*W*) were used for 2-level predictors.

To examine whether the insight effect was specific to GPT-5 output, we conducted parallel analyses on the 18 expert-generated hierarchies (6 per insight level). Safety ratings for expert hierarchies also varied significantly by level of insight (*χ*^²^_2_=6.9; *P*=.03), with absent-insight hierarchies rated lower (mean 4.72, SD 0.25) than limited-insight hierarchies (mean 5.00, SD 0.00; *P*=.03; *r*=0.67). Specificity ratings showed a similar directional pattern (*χ*^²^_2_=5.3; *P*=.07). The magnitude of the decline in safety ratings from good to absent insight was comparable across sources (*Δ*=0.25 for GPT-5 and *Δ*=0.22 for expert). Relevance, variability, and overall quality did not differ significantly by insight level for expert hierarchies (all *P*>.17).

### Blinded Review Comments

Raters provided optional free-text comments on 43 of 270 (15.9%) ratings, with 35 (81.4%) directed at AI-generated hierarchies and 8 (18.6%) at expert-generated hierarchies. As shown in Table 4, the most common theme was difficulty miscalibration, which accounted for 30 of 43 (69.8%) comments, split between exposures flagged as too difficult (n=14) and insufficiently challenging (n=16). “Too difficult” comments were concentrated among absent-insight (n=9) and limited-insight (n=4) vignettes, while “too easy” comments showed the inverse pattern, with 10 directed at good-insight vignettes. Seven comments identified missing exposure types, predominantly mirror and photograph-based exposures. At the hierarchy level, the rate of miscalibration comments did not differ significantly by source (GPT-5: 25/72, 34.7% and expert: 4/18, 22.2%; Fisher exact test *P*=.40).

**Table 4.** Blinded reviewer comments by category and hierarchy source^a^.

Category	Values, n (%)	ChatGPT (GPT-5; n=216 ratings), n	Expert (n=54 ratings), n	Representative quotes	Example of hierarchy content
Too difficult	14 (32.6)	13	1	*Might need to start with easier exposures, given the delusional beliefs.* *Concerned that for a pt with no insight some of these exposures...could be aiming too high.*	“Attend a large social event without makeup —stay for at least 1 hour, fully visible in bright lighting, no safety behaviors” (hierarchy for a 15-year-old with absent insight).
Too easy	16 (37.2)	13	3	*Could start off with harder exposures, given patient’s level of insight.* *Some of the exposures seem too brief and/or not challenging enough.*	“Briefly look in the mirror for 5 seconds, then walk away” (first item in a hierarchy for a patient with good insight).
Missing exposures	7 (16.3)	6	1	*Mirror and selfie exposures are missing.* *I would add in an exposure around pictures/selfies for greatest variability.*	A 10-item hierarchy for skin concerns containing no mirror or photograph-based exposures, focusing exclusively on social situations without camouflage.
Other	6 (14.0)	3	3	*Too many allowances for continuing to use safety behaviors.* *Exposures could be more specific.*	“Go outside wearing usual makeup” and “Skip primer while still wearing foundation” (retaining camouflage as a safety behavior within the exposure).
Total	43 (100)	35	8	—^b^	—

a Each of the 270 ratings (3 raters × 90 hierarchies) could include an optional free-text comment, of which 43 (15.9%) did. Two authors (GKJ and ACJ) independently coded each comment into 1 of 4 categories; discrepancies were resolved by consensus. Percentages reflect the proportion of the 43 comments in each category. ChatGPT (GPT-5) and Expert columns report raw comment counts; column headers indicate the number of hierarchies per source. “Too difficult” and “too easy” together constitute difficulty miscalibration (30/43, 69.8% of comments). At the hierarchy level (n=90), the rate of miscalibration comments did not differ significantly by source (Fisher exact test, *P*=.40).

b Not applicable.

### Unblinding

After unblinding, raters correctly identified the AI-generated hierarchy in 17 of 54 (31.5%) pair comparisons, which was significantly *below* chance (50%; binomial test, *P*=.009). Individual rater accuracies were 55.6% (rater 1: 10/18; *P*=.82), 33.3% (rater 2: 6/18; *P*=.24), and 5.6% (rater 3: 1/18; *P*<.001; Table 5). No pair was correctly identified by all 3 raters. Three pairs were incorrectly classified by all 3 raters, and 13 of the remaining 15 pairs were correctly identified by only 1 rater.

**Table 5.** Unblinding: rater accuracy in identifying AI-generated hierarchies^a^.

Rater	Accuracy, correct/total (%)	Binomial *P* value	Confidence^b^ (%), mean (SD)
Rater 1	10/18 (55.6)	.82	54.2 (6.7)
Rater 2	6/18 (33.3)	.24	57.2 (15.3)
Rater 3	1/18 (5.6)	<.001	57.2 (19.0)
Overall	17/54 (31.5)	.009	56.2 (14.4)

a Raters were presented with 18 pairs of ChatGPT (GPT-5)-generated and expert-generated hierarchies and asked to identify which was generated by AI. Binomial tests assessed whether accuracy differed from chance (50%).

b Confidence was rated on a 0-100 scale.

Raters reported moderate confidence in their judgments (mean 56.2%, SD 14.4%), and confidence did not differ between correct and incorrect identifications, Wilcoxon rank-sum test=337.5; *P*=.65; *r*=0.06, suggesting that raters were not reliably calibrated to distinguish between sources. Accuracy did not vary meaningfully by area of concern (27.8%‐33.3%), symptom specificity (25.9%‐37.0%), or insight (22.2%‐38.9%).

A sensitivity analysis excluding the rater with the lowest accuracy (1/18 correct, 5.6%) yielded an identification rate of 44.4% (16/36), which did not differ from chance (*P*=.62), indicating that the overall below-chance finding was driven primarily by one rater. Notably, no individual rater performed significantly above chance.

## Discussion

### Principal Findings

This study provides the first evidence that general-purpose GenAI, here, ChatGPT (GPT-5), which has not been trained or fine-tuned for specific clinical purposes, can generate exposure hierarchies for BDD that, on almost all rating dimensions assessed, received ratings as high as those created by expert clinicians. Across 4 of 5 dimensions of clinical quality assessed—relevance, specificity, safety, and overall quality—blinded raters found no significant differences between AI- and expert-generated hierarchies. However, expert-generated hierarchies were rated modestly higher on variability than AI-generated hierarchies, meaning hierarchies created by clinicians tended to provide fuller coverage across the range of exposure difficulty. These findings extend the work of Bernstein et al [28], who demonstrated similar feasibility of general-purpose GenAI (GPT-4) for creating OCD exposure hierarchies. Notably, when raters were asked to identify which hierarchy in a matched pair was AI-generated, they showed a bias toward expert-generated hierarchies, performing significantly below chance and correctly identifying the source only 31.5% of the time, although sensitivity analysis showed this was largely driven by one rater, and no rater exceeded chance accuracy. Raters were moderately confident in their judgments regardless of whether they were correct, suggesting that the AI-generated hierarchies did not contain obvious tells that distinguished them from expert work. The study’s most clinically important finding, however, concerned patient insight. Hierarchies for absent-insight cases received significantly lower safety and overall-quality ratings, and raters’ free-text comments attributed this to AI-specific difficulty miscalibration, a failure mode that the structured rating dimensions did not capture. We develop this finding, and its implications for how GenAI-generated clinical content should be evaluated, below.

### Insight

The most clinically significant finding in this study was that the level of patient insight predicted the safety and quality of AI-generated hierarchies. Hierarchies generated for patients with absent insight received lower ratings on safety and overall quality, with large effect sizes. Critically, however, this pattern was not unique to AI: safety ratings for expert-generated hierarchies also declined significantly by insight level (*P*=.03), and the magnitude of the decline from good to absent insight was comparable across sources (*Δ*=0.25 for GPT-5 and *Δ*=0.22 for experts). These parallel analyses should be interpreted cautiously, given the small number of expert hierarchies per insight level (n=6), but the convergence of the pattern across sources suggests that generating safe and appropriately calibrated exposures for patients with BDD and delusional conviction is inherently difficult, regardless of whether the author is an expert clinician or a GenAI model. On the quantitative ratings, the insight effect is thus better characterized as a shared clinical challenge than as an AI-specific limitation. However, the qualitative data tell a more nuanced story.

Where the 2 sources diverged was in the qualitative pattern of rater concerns. Of the 14 free-text comments flagging exposures as too difficult or advanced for low-insight patients, 13 were directed at AI-generated hierarchies, with raters noting that exercises would need to be broken down further and that initial exposure goals were not feasible given delusional beliefs. Only one such comment concerned an expert-generated hierarchy for the same vignettes. Taken together, the quantitative and qualitative findings suggest that, while both sources struggled with limited and absent-insight cases, expert clinicians produced better-calibrated hierarchies, for example, specifying easier initial exposures and progressing more gradually, a difference that the rating dimensions and 5-point scale were not sensitive enough to detect. The rating instrument did not include a dimension assessing whether exposure difficulty was appropriately calibrated to the patient’s clinical presentation; raters’ miscalibration concerns emerged only through the optional comment field, suggesting that a dedicated measure of difficulty appropriateness is needed to evaluate AI-generated hierarchies for complex cases. That this failure mode emerged only in the free-text comments, invisible to the structured ratings, is itself a contribution of this proof-of-concept: it identifies difficulty calibration as an essential and currently missing dimension for evaluating GenAI-generated clinical content. In this context, “less safe” appears to reflect a mismatch between the intensity of the suggested exposures and the patient’s capacity to engage with them meaningfully. For patients with absent or limited insight, this misalignment could potentially increase the risk of overwhelming distress or premature treatment dropout.

The relationship between insight and treatment outcome in BDD, however, is more nuanced than these results might initially suggest. Recent work has found that baseline insight neither predicts nor moderates symptom improvement over 24 weeks of CBT or supportive psychotherapy for BDD, concluding that although limited insight may reduce willingness to initiate treatment, it does not impede gains once patients are engaged [34]. A parallel finding has emerged in OCD, where baseline insight did not predict short-term ERP outcomes but was associated with greater severity at 12-month follow-up, and changes in insight during treatment predicted outcomes at every subsequent time point [35]. Across both studies, what mattered most was how patients’ insight changed over the course of treatment. Limited-insight patients can benefit from well-executed ERP [28,34], which raises the clinical stakes of getting the hierarchy right.

What makes this calibration difficult is that insight in BDD does not operate like other clinical variables do. Research has shown that insight’s connections to other symptoms fluctuate unpredictably over the course of treatment, and that its improvement emerges indirectly as a downstream consequence of behavior change rather than as a direct treatment target [36]. Effective exposure planning for low-insight patients depends on anticipating how the patient will relate to the tasks, whether motivational groundwork is needed, and whether the difficulty levels risk reinforcing avoidance, all of which are contextual judgments that emerge from the therapeutic relationship over time and that a single-turn prompt cannot replicate.

This limitation illustrates what Li et al [37] term contextual errors: outputs that appear clinically plausible but miss critical patient-specific information. General-purpose GenAI received ratings comparable to expert-created hierarchies for straightforward, vignette-based presentations but did not adequately adjust for patients with delusional conviction associated with absent insight. In real-world practice, hierarchy construction must also account for relevant BDD factors not represented in the simulated vignettes, including psychiatric comorbidities [38], prior suicidal thoughts or behaviors, and treatment history [6], and culturally specific appearance concerns [39], all of which influence which exposures are appropriate and how their difficulty is graded across ERP exercises. Also, the therapeutic alliance itself informs calibration: clinicians adjust the hierarchy in real-time based on the patient’s emotional responses, engagement, and progress across sessions. These contextual demands are unlikely to be addressed by “zero-shot” prompting, though iterative or multi-turn approaches may narrow this gap.

### Comparison to Prior Work

The present findings replicate and extend our prior work evaluating the role of GenAI in clinician-facing tools [28]. Both studies used the same general design, expert-generated benchmarks, systematically varied simulated vignettes, and blinded clinician ratings. In both studies, the AI-generated hierarchies were rated as safe and clinically acceptable. However, the 2 studies diverged in ways that illuminate important differences in the application of GenAI for generating exposure hierarchies for OCD compared with BDD.

The biggest difference concerns distinguishability. In the OCD study, blinded raters correctly identified AI-generated hierarchies at rates well above chance (67%‐94% across raters), and expert-generated hierarchies were rated significantly higher on appropriateness, specificity, variability, and overall usefulness. However, in this study, raters identified AI-generated hierarchies at rates significantly below chance (17/54, 31.5%), consistently misidentifying them as clinician generated. A sensitivity analysis excluding the rater with the lowest accuracy (1/18 correct) yielded a nonsignificant identification rate of 44.4% (16/36), suggesting that the below-chance finding was driven primarily by one rater rather than reflecting a consistent pattern across all evaluators. One possibility is that raters held relatively pessimistic expectations about how well GenAI could perform on this clinically complex task; expecting, for instance, that GenAI would produce lower-quality, less nuanced, or less clinically grounded ERP suggestions compared to expert clinicians. If so, raters may have used perceived quality as a proxy for hierarchy source. Consistent with this, surveys of mental health professionals have found generally low expectations of AI in treatment-delivery roles, although practitioners with cognitive behavioral orientations, as our raters were, tend to be somewhat more receptive than those from other traditions [40]. Interestingly, because no significant differences emerged between sources on relevance, specificity, safety, or overall quality, and the only dimension on which clinician hierarchies were rated higher was variability (with a small effect), the heuristic (that AI would produce worse hierarchies than experts) would have paradoxically led raters to misidentify the more formulaic or evenly structured hierarchy as clinician-generated. These results suggest that clinicians’ intuitions about what AI-generated clinical content looks like may not keep pace with the capabilities of current models. That blinded specialists could not reliably distinguish the 2 sources, on a task as clinically nuanced as BDD hierarchy construction, is notable given that the same paradigm applied to OCD with GPT-4 produced large source differences (ds>1 on 4 of 5 dimensions) and 67% to 94% identification accuracy [28]. At the same time, indistinguishability is not the central contribution of this study; the more informative result is where the model fell short, namely, difficulty calibration for low-insight cases. Similarly, the rate of difficulty-related comments did not differ significantly between GPT-5 and expert hierarchies (*P*=.40), though this likely reflects limited power rather than true equivalence, given that 86.7% (26/30) of miscalibration comments were directed at GPT-5 output.

Several factors may contribute to this greater indistinguishability. One possibility is model advancement: this study used GPT-5, whereas Bernstein et al [28] used GPT-4. However, because the studies also differed in clinical domain, vignette set, rater pool, and rating instrument, and because this study did not directly compare multiple model versions, the contribution of model advancement cannot be isolated from these other differences. Nonetheless, task characteristics likely also played a role. BDD exposure hierarchies tend to involve a more limited set of behavioral domains—such as social situations, mirror and photograph-based exposures, and reduction of camouflaging and checking—compared to the heterogeneity of OCD subtypes, which range from contamination to harm to sexual and religious obsessions and may require imaginal exposures, scripted provocations, and other modality-specific tasks. This relative homogeneity may make BDD hierarchies more tractable for a language model to approximate. Conversely, Bernstein et al [28] found that the model for OCD struggled most by omitting imaginal exposures, generating insufficient ritual prevention instructions, and failing to deviate from vignette-provided examples, errors that reflect the broader behavioral repertoire required for OCD. In this study, the analogous gap was the omission of mirror and photograph-based exposures, which raters flagged in 6 comments directed at GPT-5 hierarchies. Whether this reflects a true limitation or an appropriate separation of in vivo and perceptual retraining components [41] warrants further consideration, but it represents a potentially more addressable limitation.

### Limitations

Several limitations should be considered. All hierarchies were generated from simulated vignettes rather than real patients, who may present with comorbidities, treatment history, suicide risk, culturally specific appearance concerns, and an evolving therapeutic relationship. Although the simulated vignettes had high face validity and were based on prototypical clinical presentations, they lacked the clinical complexity of many patients and therefore these results may not generalize to real-world clinical populations. Relatedly, the 10-item hierarchy was generated through a “zero-shot” prompting process, where no prior examples or additional feedback was provided to shape the initial AI-generated ERP hierarchy; however, in clinical practice, exposure hierarchies are dynamic working documents that evolve as the therapist learns more about the patient’s responses over the course of treatment. Ratings also showed a restricted range at the upper end of the scale, with 258 (95.6%) to 269 (99.6%) of 270 ratings falling between 4 and 5. This likely limited sensitivity to fine-grained quality differences, and the rating dimensions did not include a measure of difficulty calibration, which was the most frequently identified shortcoming in the qualitative comments. Although the 5 rating dimensions were rationally derived by content experts and published in prior work, they have not yet been formally validated. For instance, we did not subject the rating dimensions to independent expert review to establish their face validity. A formal validation of an instrument for evaluating GenAI-generated exposure hierarchies is an important direction for future work. Raters likewise did not undergo formal calibration before rating, so the high interrater agreement may partly reflect shared default rating tendencies rather than fully independent convergence. An imbalance between the expert benchmark of 18 hierarchies and 72 AI-generated hierarchies may have limited statistical power, making it challenging to identify small but perhaps meaningful differences between the hierarchy sources. In addition, because this study changed both the clinical domain (OCD to BDD) and the model version (GPT-4 to GPT-5) relative to Bernstein et al [28], improvements in AI-generated ERP hierarchies cannot be attributed to either factor in isolation. Furthermore, the AI-generated hierarchy was always presented in the top position during unblinding, and a sensitivity analysis showed the below-chance result was driven largely by one rater (16/36, 44.4% accuracy without that rater; *P*=.62), so the unblinding findings should be interpreted cautiously. Lastly and most critically, this study assessed the perceived quality rather than clinical effectiveness of ERP hierarchies; high expert ratings per se do not establish that these hierarchies would produce therapeutic benefit when implemented in treatment.

### Conclusions

Several lines of investigation follow from these results. First, clinical implementation studies are needed to test whether therapists can effectively use AI-generated hierarchies with real patients and whether doing so improves efficiency without compromising treatment outcomes. Second, “multiturn” or sequential, iterative prompting, in which the model receives feedback and revises its output, may address some of the limitations observed here, particularly the miscalibration of difficulty level for low-insight cases. Third, targeted vignette modifications specifying required exposure modalities (eg, mirror and photograph exposures) could address the most common content gaps identified by raters. Fourth, systematic testing of different AI tools and model versions, holding clinical domain and rating instrument constant, would clarify whether the differences observed between our OCD and BDD studies reflect model advancement, task characteristics, or both. Finally, future work should develop more discriminating evaluation rubrics, including a dedicated “difficulty calibration” dimension that indexes whether the difficulty and intensity of exposure exercises are appropriately graded to the patient’s clinical presentation, particularly their level of insight and capacity for engagement. Scales with more gradations (eg, a 10-point scale) and behaviorally anchored descriptors may further improve discrimination at the upper end of the scale and guard against ceiling effects.

These findings support the potential of AI-assisted hierarchy generation as a clinician-facing augmentation tool, particularly for addressing the well-documented shortage of BDD-trained providers. AI-generated hierarchies could serve as an initial draft that clinicians refine based on their clinical judgment and knowledge of the patient, but they should not be treated as ready-to-implement treatment plans without review. Although this study did not measure time or workload, generating a clinically acceptable ERP hierarchy is time-consuming. Thus, using AI to generate a draft in a fraction of this time may have implications for efficiency in treatment planning, a hypothesis that warrants direct investigation. Crucially, these findings suggest that AI-assisted hierarchy development is best suited for patients with at least partial insight. For individuals with limited or absent insight, particularly when delusional conviction or elevated suicide risk is present, clinician oversight is essential to verify that exposure difficulty is appropriately calibrated. AI-assisted hierarchy tools may be especially valuable for clinicians with limited experience in BDD-specific ERP or in settings where expert consultation is scarce, provided that systematic insight assessment and safety checks are incorporated into the clinical workflow. It is also worth acknowledging that patients may already be using general-purpose GenAI to generate or modify exposure content independently, outside of therapeutic contexts. Survey data indicate that roughly a quarter of community respondents, most of whom reported prior mental health difficulties, have used AI tools for mental health purposes, most commonly for immediate support during distress or as a substitute for a therapist [42]. This emphasizes the importance of understanding both the capabilities and limitations of AI-generated hierarchies across the full range of clinical presentations.

## Supplementary material

10.2196/98393 Multimedia Appendix 1 Part A presents example simulated patient vignettes, including the low- and high-symptom-specificity versions of a single case, to illustrate how the standardized prompt template was applied across the factorial design. Part B presents the blinded rating rubric, including the exact item wording and response scales for each of the 5 quality dimensions (relevance, specificity, variability, safety, and overall quality) as displayed to raters in the REDCap survey.

10.2196/98393 Checklist 1 STROBE checklist.

## Abbreviations

AC2: Gwet agreement coefficient 2
BDD: body dysmorphic disorder
CBT: cognitive behavioral therapy
*DSM-5*: *Diagnostic and Statistical Manual of Mental Disorders, Fifth Edition*
ERP: exposure and response prevention
GenAI: generative AI
IRB: Institutional Review Board
OCD: obsessive-compulsive disorder
STROBE: Strengthening the Reporting of Observational Studies in Epidemiology
