# Supplementary material for: AI-Generated Exposure Hierarchies for Body Dysmorphic Disorder: Proof-of-Concept Study
Source: JMIR Form Res. 2026 Nov 3;10:e98393. doi: 10.2196/98393 (PMC13730212; doi:10.2196/98393)
Supplement: Supplementary file 1 [file formative-v10-e98393-s002.pdf]

Multimedia Appendix 2. STROBE Checklist

Section/Topic	Item No	Recommendation	Reported on Page/Section
Title and abstract	1	(a) Indicate the study's design with a commonly used term in the title or the abstract	Title includes "Proof-of-Concept Study"; Abstract states study design.
	1	(b) Provide in the abstract an informative and balanced summary of what was done and what was found	Abstract, Background through Conclusions.
**INTRODUCTION**			
Background/rationale	2	Explain the scientific background and rationale for the investigation being reported	Introduction, paragraphs 1–4.
Objectives	3	State specific objectives, including any prespecified hypotheses	Introduction, final paragraph.
**METHODS**			
Study design	4	Present key elements of study design early in the paper	Methods, Data Analysis.
Setting	5	Describe the setting, locations, and relevant dates, including periods of recruitment, exposure, follow-up, and data collection	Methods, Ethics Approval and Hierarchy Generation (data collection dates, institutional setting).
Participants	6	(a) Give the eligibility criteria, and the sources and methods of selection of participants	N/A, no human participants recruited. Study used simulated vignettes. Expert raters described in Methods, Blinded Review.
	6	(b) For matched studies, give matching criteria and number of exposed and unexposed. For cross-sectional studies, describe methods of ascertainment	Methods, Hierarchy Generation, 72 vignettes from complete factorial design; Human Expert Hierarchies, 18 vignettes, one per cell.
Variables	7	Clearly define all outcomes, exposures, predictors, potential confounders, and effect modifiers	Methods, Blinded Review, five rating dimensions defined; Initial Review, task completion and information integration defined; Hierarchy Generation, five vignette characteristics defined.
Data sources/measurement	8	For each variable of interest, give sources of data and details of methods of assessment	Methods, Blinded Review, rating scale described; Initial Review, coding described. Full item wording in Multimedia Appendix 3.
Bias	9	Describe any efforts to address potential sources of bias	Methods, Blinded Review, blinding procedure; Limitations, position confound and sensitivity analysis.
Study size	10	Explain how the study size was arrived at	Methods, Hierarchy Generation, complete 3×3×2×2×2 factorial yielded 72 vignettes; Human Expert Hierarchies, 18 (one per body area × insight × specificity cell).
Quantitative variables	11	Explain how quantitative variables were handled in the analyses	Methods, Data Analysis, nonparametric tests; hierarchy-level means computed by averaging across 3 raters.
Statistical methods	12	(a) Describe all statistical methods, including those used to control for confounding	Methods, Data Analysis, Kruskal-Wallis, Wilcoxon, Fisher's exact, binomial tests, Gwet's AC2 described.
	12	(b) Describe any methods used to examine subgroups and interactions	Methods, Blinded Review, subgroup analyses by vignette characteristics; parallel analyses on human hierarchies.
	12	(c) Explain how missing data were addressed	N/A, no missing data; all 270 ratings complete.
	12	(d) If applicable, explain how loss to follow-up was addressed	N/A, cross-sectional design.
	12	(e) Describe any sensitivity analyses	Results, Unblinding, sensitivity analysis excluding lowest-accuracy rater.
**RESULTS**			
Participants	13	(a) Report numbers of individuals at each stage of study	Results, Initial Review, 69/72 complete; Blinded Review, 90 hierarchies rated (270 ratings total).

	13	(b) Give reasons for non-participation at each stage	N/A, all vignettes generated output; all hierarchies rated.
	13	(c) Consider use of a flow diagram	N/A, straightforward flow; all hierarchies rated by all raters.
Descriptive data	14	(a) Give characteristics of study participants and information on exposures and potential confounders	Methods, Blinded Review — rater characteristics (years post-degree, practice settings, BDD caseload, ERP usage).
	14	(b) Indicate number of participants with missing data for each variable of interest	N/A, no missing data.
Outcome data	15	Cross-sectional study, Report numbers of outcome events or summary measures	Results, Blinded Review, means, SDs, ranges for all dimensions; Tables 2 and 3.
Main results	16	(a) Give unadjusted estimates and, if applicable, confounder-adjusted estimates and their precision	Results, Blinded Review, Wilcoxon tests with rank-biserial correlations and P values; Table 3.
	16	(b) Report category boundaries when continuous variables were categorized	N/A, categorical predictors used as defined by factorial design.
	16	(c) If relevant, consider translating estimates of relative risk into absolute risk	N/A.
Other analyses	17	Report other analyses done—eg analyses of subgroups and interactions, and sensitivity analyses	Results, parallel insight analyses on human hierarchies; sensitivity analysis excluding Rater 3; Fisher's exact test on comment patterns; Gwet's AC2.
**DISCUSSION**			
Key results	18	Summarise key results with reference to study objectives	Discussion, Principal Findings.
Limitations	19	Discuss limitations of the study, taking into account sources of potential bias or imprecision	Discussion, Limitations.
Interpretation	20	Give a cautious overall interpretation of results considering objectives, limitations, multiplicity of analyses, results from similar studies, and other relevant evidence	Discussion, Insight and Comparison to Prior Work sections.
Generalisability	21	Discuss the generalisability (external validity) of the study results	Discussion, Limitations, simulated vignettes vs. real patients; Conclusions, clinical boundaries.
**OTHER INFORMATION**			
Funding	22	Give the source of funding and the role of the funders for the present study	Funding statement.
