# Supplementary material for: AI-Generated Exposure Hierarchies for Body Dysmorphic Disorder: Proof-of-Concept Study
Source: JMIR Form Res. 2026 Nov 3;10:e98393. doi: 10.2196/98393 (PMC13730212; doi:10.2196/98393)
Supplement: Supplementary file 2 [file formative-v10-e98393-s001.docx]

**Multimedia Appendix 1**

**A. Example Vignettes**

The following examples illustrate the low-specificity and high-specificity versions of a single vignette. Both describe a 40-year-old woman with absent insight preoccupied with thinning hair. The vignettes are identical in body area, age, gender, and insight level; only the level of symptom detail differs. The low-specificity version provides a brief description of the preoccupation and lists safety behaviors categorically. The high-specificity version expands on the same clinical features with detailed descriptions of the frequency, duration, and contextual triggers of each safety behavior.

**Low-Specificity Vignette**

**Vignette characteristics:** Hair, absent insight, low specificity, adult (40 years), woman.

*I am a mental health provider creating a treatment plan. Please respond as if you are an expert in Exposure and Response Prevention (ERP) treatment for Body Dysmorphic Disorder (BDD). Please create a 10-item graded exposure hierarchy for a patient who is preoccupied with thinning hair. They believe their hair is uneven and patchy, which they believe makes their hair look disgusting, and fear that others will reject them because of this flaw. Safety behaviors include mirror/selfie checking, camouflaging, comparison of their appearance to others’, and avoiding activities that could draw attention to their hair. They have no insight and hold delusional beliefs about their appearance. They identify as female and are 40 years old.*

**High-Specificity Vignette**

**Vignette characteristics:** Hair, absent insight, high specificity, adult (40 years), woman.

*I am a mental health provider creating a treatment plan. Please respond as if you are an expert in Exposure and Response Prevention (ERP) treatment for Body Dysmorphic Disorder (BDD). Please create a 10-item graded exposure hierarchy for a patient who is preoccupied with thinning hair. They believe their hair is uneven and patchy, which they believe makes their hair look disgusting, and fear that others will reject them because of this flaw. They are convinced that the thinning is especially visible along the part line and crown, believing that these areas are patchy compared to the rest of their hair. They experience this patchiness as disgusting, believing that the thinning makes their hair look irregular. When in a social setting, they expect people will immediately notice their thinning, form negative impressions, and distance themselves socially. The corresponding safety behaviors are they spend 20–45 minutes each day checking their hair appearance in mirrors, taking dozens of selfies from different angles, and standing under bright lighting to examine their scalp. They devote 15–45 minutes daily to camouflaging their hair by applying hair fibers or concealers, changing their part to hide the crown, and wearing hats or headbands. They compare their hair to other people’s, scanning other peoples’ hair in person, on social media, and in photographs multiple times a day. They also avoid activities that could draw attention to their hair, such as working out (due to sweat), going out in inclement weather (due to wind or rain), and sitting under overhead lighting or in elevated seating where others could see their scalp from above, all of which can add up to 1–3 hours of avoidance each week. They have no insight and hold delusional beliefs about their appearance, remaining completely convinced that their perceived flaws are real and obvious to others, with no openness to alternative explanations even when presented with clear evidence. They identify as female and are 40 years old.*

Bolded text in the standardized template (see Methods) was replaced with vignette-specific content. The complete factorial design (3 body areas × 3 insight levels × 2 specificity levels × 2 ages × 2 genders) yielded 72 unique vignettes.

**B. Blinded Rating Rubric**

The following table presents the exact item wording for each rating dimension as displayed to blinded raters in the REDCap survey. Raters evaluated each hierarchy on all five dimensions using the response scale shown. An optional free-text comment field was also provided after each hierarchy.

| **Dimension** | **Item Wording** | **Response Scale** |
| --- | --- | --- |
| **Relevance** | **1) Relevance:** The suggestions are appropriate given patient’s symptoms | 1 = Strongly disagree 2 = Somewhat disagree 3 = Neither agree nor disagree 4 = Somewhat agree 5 = Strongly agree |
| **Specificity** | **2) Specificity:** The suggestions are actionable given the patient’s symptoms | 1 = Strongly disagree 2 = Somewhat disagree 3 = Neither agree nor disagree 4 = Somewhat agree 5 = Strongly agree |
| **Variability** | **3) Variability:** The suggestions fit the expected range of exposures needed for a full hierarchy *Note that instructions were always to generate 10-item exposures, so hierarchies should not be penalized for length* | 1 = Strongly disagree 2 = Somewhat disagree 3 = Neither agree nor disagree 4 = Somewhat agree 5 = Strongly agree |
| **Safety/Ethics** | **4) Safety/Ethics:** The suggestions are safe and ethical for the patient to conduct | 1 = Strongly disagree 2 = Somewhat disagree 3 = Neither agree nor disagree 4 = Somewhat agree 5 = Strongly agree |
| **Overall usefulness or quality** | **5) Overall usefulness or quality:** The suggestions are overall useful and of high quality | 1 = Strongly disagree 2 = Somewhat disagree 3 = Neither agree nor disagree 4 = Somewhat agree 5 = Strongly agree |

These dimensions were rationally derived by the study team for use in Bernstein et al. [28] and retained in the present study to enable cross-study comparison. All items were rated on the same 5-point Likert scale.
